# Death from Hemorrhage

**Published:** 1867-03

**Authors:** G. B. Lester

**Affiliations:** Late Surgeon 6th U.S.V. Inf’y, of Oswego, Ill.


*Death from Hemorrhage.* By G. B. LESTER, M.D., late Surgeon 6th U.S.V. Inf'y, of Oswego, Ill.

In January, 1866, I was stationed at Denver, C.T., as Chief Medical-Officer, District of Colorado, and among the many friends made there, I found none more genial and hospitable, nor one who stood higher in his profession and as a gentleman, than the subject of the present communication. A few months previously, he had been chosen State Senator, and everything promised fair for a long, honorable, and useful life.

On the 10th of January, 1866, L. B. McLain, M.D., came to my office with a punctured wound, half an inch in extent, situated in front of the metacarpal bone of the thumb, and on a line parallel with it, passing from above downwards and backwards striking the metacarpal bone. Hemorrhage, slight and arterial, but easily controlled. I thought him very much excited from so small an injury, when he informed me that his brother had lost his life from hemorrhage following the extraction of a tooth, and he feared the same result. I saw him on the 11th. There had been no further hemorrhage; he was feeling well and was attending to his professional duties.

On the night of the 14th, he sent for me, as hemorrhage had set in, none having taken place since I saw him on the 11th. The edges of the wound were everted, and blood was oozing from it. The whole cut surface was lighter colored than natural and "frothy," hard, slightly swollen, and painful. I applied a compress and roller, suppressing hemorrhage, and left him comfortable.

*15th.* Sent for me before I was out of bed this morning. The wound becoming very painful in the night, he had removed the dressing and allowed it to bleed. After consultation with

Drs. Buckingham and Morrison, styptics, compress, and bandage were again applied, and graduated compression on the radial and ulnar arteries above the wrist. Pain was controlled with opium. No hemorrhage occurred now for a week, but the wound continued to enlarge and discharged a thin, sanious pus. The limb, for some distance above the wrist, became much swollen and œdematous, and not a trace of granulation or effort at repair could be discovered in the wound.

*22d.* Hemorrhage again set in towards afternoon, and after trying every appliance and making exploration of the hand, with a view of ligating any arteries we might discover, it was found useless.

The next day, *23d*, I ligated the radial and ulnar arteries just above the wrist, with the effect of checking the hemorrhage for about twenty-four hours. It returned on the *24th*, and, after consultation with Dr. Buckingham and others, amputation was the only means that seemed to offer the least possible chance for a favorable issue. Accordingly, amputation was performed by Dr. Buckingham, and every care taken in ligating the arteries. Reäction was well established after amputation.

*25th.* Hemorrhage again set in this morning from the stump. The flaps reöpened and the blood was oozing from a few arterial branches, almost capillary in their minuteness. I took them up and included them in a ligature; left the flaps open for an hour, when, everything appearing favorable, I closed them, hoping that all would now be well; but that evening there was oozing of blood, which was checked by pounded ice kept in close apposition with the stump.

*29th.* Stump was dressed. No adhesions had taken place in any part of the flap, but a dark, grumous clot caused it to bulge out and protruded itself beyond the surface. This was trimmed off even with the surface, and the stump was thoroughly cleansed.

*31st.* Stump again dressed; appearances but slightly changed; the clot beginning to break down. An injection of alum, ʒj. to ℥j. of water, was made between the flaps.

*Feb'y 1st.* Appetite failing, and much debility. Continued in this way, refusing all food or medicine, until the morning of the 2d Feb'y, at 4 o'clock when he ceased to exist.

Appearances of the stump after death, showed disorganization of the tissues for three inches above the point of amputation, leaving absolutely nothing but the skin and superficial fascia covering the bone. From that point, the finger would pass readily to near the elbow-joint. Drs. Buckingham, Strode, McClellan, and Gehrung were associated with me, from time to time, in the treatment of the case, and to them I was much indebted for advice and assistance.

