# Erratum

**Published:** 1867

**Authors:** 


*Erratum.*—By an oversight of the proof reader of the March number of the JOURNAL, the case reported by G. B. Lester, M.D., (p. 112) in the third line of the third paragraph should, but does not, commence and read as follows:

"The edges of the wound were everted, and blood was oozing from *the* whole cut surface, lighter colored than natural and frothy; *hand* slightly swollen and painful."

The italicised words indicate the needed corrections.

